# Long-Range and Dead-Zone-Free Dual-Comb Ranging for the Interferometric Tracking of Moving Targets

**DOI:** 10.1021/acsphotonics.4c02199

**Published:** 2025-03-21

**Authors:** Sandro L. Camenzind, Lukas Lang, Benjamin Willenberg, Justinas Pupeikis, Hayk Soghomonyan, Robert Presl, Pabitro Ray, Andreas Wieser, Ursula Keller, Christopher R. Phillips

**Affiliations:** † Department of Physics, Institute for Quantum Electronics, ETH Zurich, 8093 Zurich, Switzerland; ‡ Department of Civil, Environmental and Geomatic Engineering, Institute of Geodesy and Photogrammetry, ETH Zurich, 8093 Zurich, Switzerland

**Keywords:** metrology, dual-comb, ranging, distance, dead zone, real-time, accuracy, precision

## Abstract

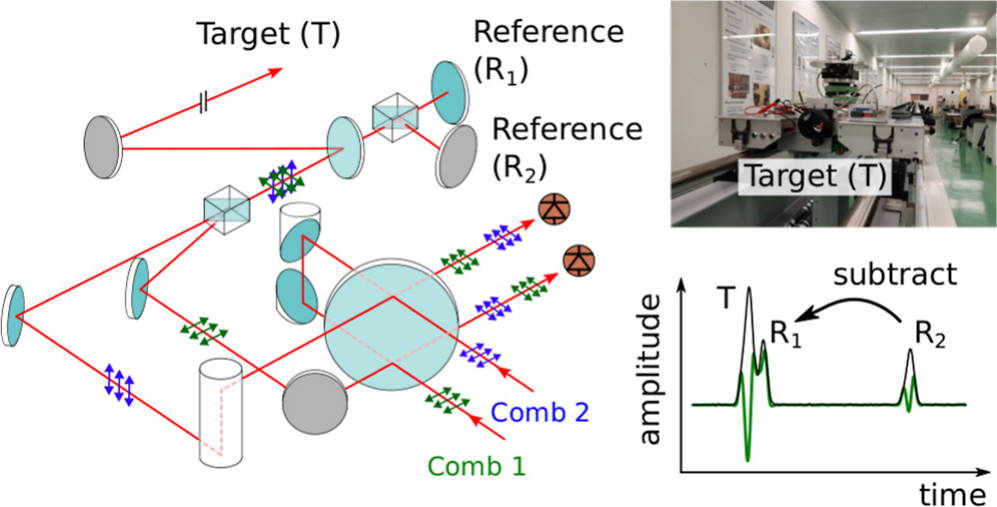

Dual-comb ranging has emerged as an effective technology for long-distance metrology, providing absolute distance measurements with high speed, precision, and accuracy. Here, we demonstrate a dual-comb ranging method that utilizes a free-space transceiver unit, enabling dead-zone-free measurements and simultaneous ranging with interchanged comb roles to allow for long-distance measurements, even when the target is moving. It includes a graphics processing unit (GPU)-accelerated algorithm for real-time signal processing and a free-running single-cavity solid-state dual-comb laser with a carrier wavelength λ_c_ ≈ 1055 nm, a pulse repetition rate of 1 GHz, and a repetition rate difference of 5.06 kHz. This combination offers a fast update rate and sufficient signal strength to reach a single-shot time-of-flight precision of around 0.1 μm (i.e., <λ_c_/4) on a cooperative target placed at a distance of more than 40 m. The free-running laser is sufficiently stable to use the phase information for interferometric distance measurements, which improves the single-shot precision to <20 nm. To assess the ranging accuracy, we track the motion of the cooperative target when moved over 40 m and compare it to a reference interferometer. The residuals between the two measurements are below 3 μm. These results highlight the potential of this approach for accurate and dead-zone-free long-distance ranging, supporting real-time tracking with nm-level precision.

## Introduction

Long-range absolute distance measurements are crucial for a wide range of application fields, e.g., satellite navigation and formation flying,^1,2^ surveying, geodesy, and mapping,^3−5^ autonomous driving,^6−8^ and the precision manufacturing industry.^9−11^ The demanding requirements of these applications in terms of speed, range, precision, and accuracy propelled the development of various techniques for absolute distance measurements such as pulsed time-of-flight (ToF) light detection and ranging (LiDAR),^12,13^ amplitude-modulated continuous-wave LiDAR,^5,14^ frequency-modulated continuous-wave LiDAR,^15,16^ and synthetic wavelength interferometry.^17,18^

Femtosecond pulsed lasers and optical frequency combs^19−22^ enable boosting the performance of conventional ranging technologies^23−26^ and the development of new techniques such as dual-comb ranging.^27−30^ Dual-comb ranging is particularly interesting for high-precision applications at long-range, as it can combine absolute distance information (from a ToF-based measurement) with the high precision of interferometric measurements. This technique is based on linear optical sampling of a signal pulse train (which probes the distance between a reference and target plane) with a local oscillator pulse train.^27^ This sampling is enabled by the pulse repetition rate difference Δ*f*_rep_ between the combs. It leads to a down-conversion of the optical time and frequency domain information to the more accessible electronic domain, which allows for precise measurements of the distance between reference and target.

With a source that exhibits a high pulse repetition rate *f*_rep_, the dual-comb ranging approach also offers a fast update rate (determined by Δ*f*_rep_), which is limited by the aliasing condition to Δ*f*_rep_ < *f*_rep_^2^/(2Δν_opt_) for a given Δν_opt_ (full optical bandwidth shared by the two combs). For example, a pair of 100 GHz silicon-nitrite microring resonators enabled an ultrahigh update rate of 96 MHz.^31^ However, the high repetition rate leads to a reduced nonambiguity range (NAR) in distance measurements. The NAR is given by *R*_A,*i*_ = υ_g_/(2*f*_rep,*i*_), where the index *i* = 1, 2 indicates the signal comb used to sample the distance between the reference and target plane, and υ_g_ is the group velocity. For a 100 GHz dual-comb system, *R*_A,*i*_ is around 1.5 mm in ambient air.

Established dual-comb generation platforms based on femtosecond solid-state and fiber lasers with repetition rates of around 100 MHz offer a much larger NAR. However, such lasers do not support a high update rate unless with a considerably narrowed optical bandwidth^32^ or alternative detection schemes leading to reduced single-shot precision^33^ or increased system complexity.^30^ This highlights the challenge of combining long-distance measurements with high precision and fast update rates to track moving objects.

To address this, we utilized a low-noise 1 GHz solid-state dual-comb with a repetition rate difference of Δ*f*_rep_ = 5.06 kHz as this provides a reasonably fast update rate for tracking moving targets while maintaining a broad bandwidth and large photon number per pulse to enable high single-shot measurement precision. This laser source is combined with a free-space transceiver unit which avoids problematic spurious ghost pulses, enables dead-zone-free measurements at all distances by creating two slightly delayed reference reflections, and allows to measure the distance simultaneously with the role of the two combs interchanged to extend the nonambiguity range using the Vernier effect,^27^ even for targets in motion.^34^

To leverage the high measurement update rate, we developed a graphics processing unit (GPU)-accelerated data acquisition platform to process the acquired electronic signals, extract the ToF distance, and perform phase-based interferometric ranging in real-time to enable continuous tracking of the target’s position with interferometric precision. For this application, the GPU offers faster processing speeds compared to a central processing unit (CPU), particularly for computations such as the fast Fourier transform (FFT), which are well-suited for parallelization. Furthermore, GPU-based approaches are appealing for dual-comb data processing since they combine real-time processing capabilities with a convenient programming interface.^35,36^

The dual-comb ranging setup, including the data analysis routine, is discussed in the next section. To assess the accuracy and precision of the dual-comb ranging system, we track the motion of a movable trolley on a linear comparator bench over a distance of 40 m, which is discussed in the subsequent section. Finally, we summarize the key results of this work.

## Experimental Setup

### Theoretical Background on ToF Precision

The precision of the dual-comb interferogram (IGM) arrival time estimation can be formulated in terms of a more general ToF estimation framework as follows: the Cramer-Rao lower bound (CRLB) for the arrival time of a pulse in a ranging measurement subject to additive white noise is summarized in ref (37). Adapting this bound for the case of dual-comb ranging under the assumption that the measurement is shot-noise-limited and that the local oscillator has a much higher average power than the returning signal comb, we find a simple approximate lower bound on the time-of-flight variance due to the additive noise floor
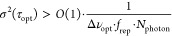
1where *N*_photon_ is the effective number of detected photons of the returning signal beam, and *O*(1) represents a pulse-shape-dependent prefactor. It has been shown that direct ToF calculations can readily be used to obtain μm-level precision.^27−29^ Obtaining precise ToF estimates is particularly crucial to leverage phase information in the IGMs. If the ToF can be reliably determined to better than λ_c_/4, the distance estimate can be refined by the much more sensitive IGM phase measurements.^27^

Equation 1 shows how gigahertz repetition rate lasers with a broad optical bandwidth are preferable in terms of ToF precision. However, another relevant consideration is the NAR. As discussed in ref (27), when the target is measured simultaneously with the role of the combs interchanged, it is possible to infer the number of nonambiguity ranges to the target without recourse to a separate measurement. To accomplish this, it is necessary to determine the ToF to within one-half of the optical delay step between subsequent pairs of pulses, which is given by
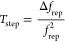
2

Few-gigahertz lasers represent a sweet spot where a realistic ToF precision is sufficient for both interferometric hand-over and nonambiguity range unwrapping while also offering fast (kilohertz) update rates. For example, a 1 GHz dual-comb with 3 THz optical bandwidth, a repetition rate difference of Δ*f*_rep_ = 5 kHz, and a wavelength of 1 μm requires a ToF estimation to within 2.5 fs for nonambiguity range estimation and 1.7 fs for interferometric hand-over. Applying these values to eq 1 assuming a 1 μW returning power and a photodiode responsivity of 0.7 A/W yields a CRLB of 0.6 fs, i.e., sufficient for both steps assuming peak finding at the CRLB limit.

Based on the above considerations, gigahertz solid-state lasers are compelling sources for precise and long-distance dual-comb ranging, as they offer kilohertz measurement update rates, multi-THz bandwidths, low-noise properties, and a direct free-running oscillator-based setup with minimal complexity and substantial average power. We demonstrate this potential via a novel dual-comb laser source, a new transceiver unit design, and a real-time data processing routine.

### Laser Source

**Figure 1:**
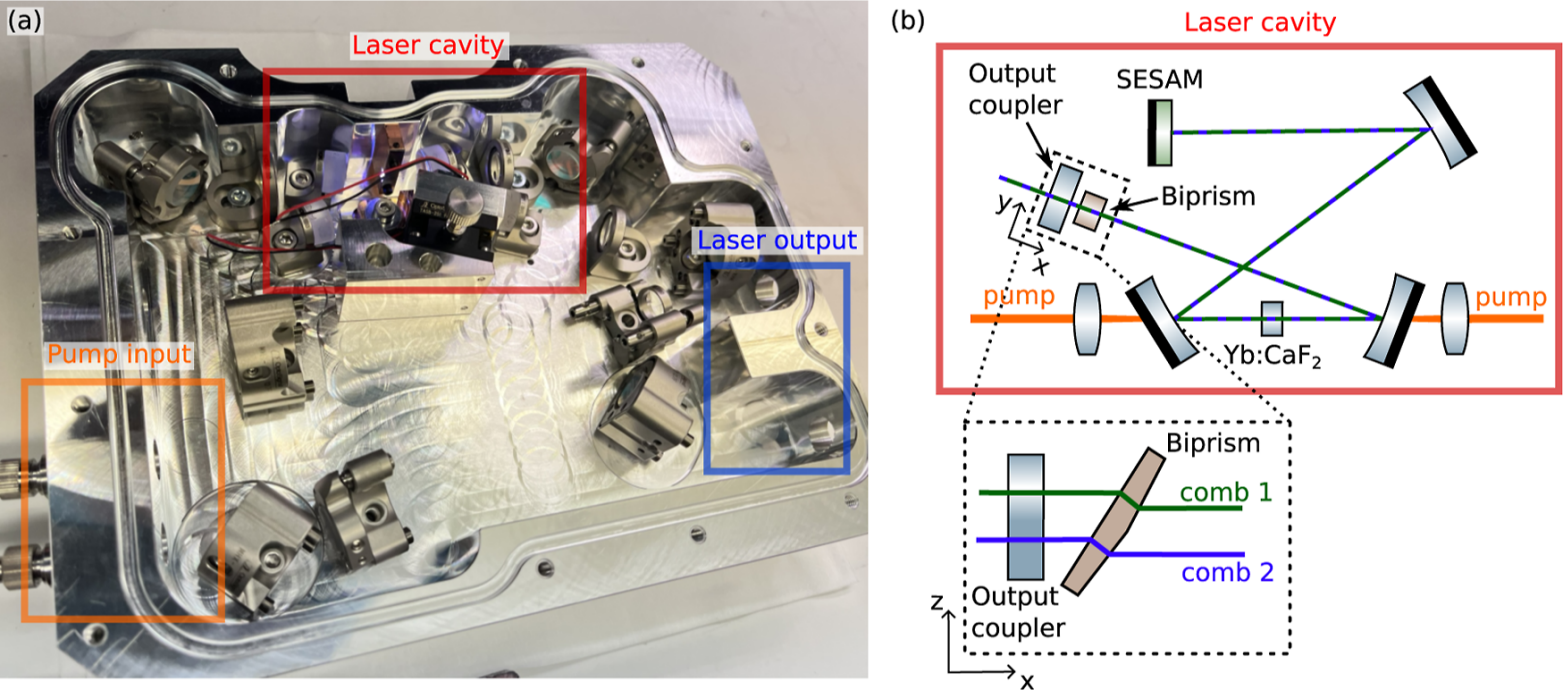
(a) Picture of the laser in a prototyped housing. The pump input is highlighted in orange, the laser cavity in red, and the laser output in blue. Not shown in this image are the fiber collimation units which were later installed in the laser output to couple the light into single-mode fibers. (b) Schematic of the bow-tie laser cavity with an Yb:CaF_2_ gain crystal, a biprism in transmission with an apex angle of 177° for spatial multiplexing, a semiconductor saturable absorber mirror for soliton modelocking and an output coupler with a transmission of 0.8%.

The laser source is a gigahertz single-cavity dual-comb laser conceptually similar to the one presented in ref (38). The laser is shown in Figure 1a with an open lid. It uses single-mode diode pumping and a spatially multiplexed cavity^39^ based on a biprism in transmission as shown in Figure 1b. The simulated evolution of the intracavity mode is shown in the Supporting Information. Compared to the Yb:CALGO laser in ref (38), the main modifications are (i) the use of a different gain medium, Yb:CaF_2_; (ii) the use of two pump diodes instead of one; and (iii) the integration of the laser into a robust prototype setup. The Yb:CaF_2_ gain material is beneficial in the context of low-noise lasers since it has a lower nonlinear refractive index than Yb:CALGO^40,41^ and offers a small emission cross section, which enables a low relaxation oscillation frequency and therefore strong low-pass filtering of pump intensity noise.^42^

The laser operates in the soliton modelocking regime^43^ with a nominal round-trip group delay dispersion of −150 fs^2^, and modelocking is obtained by a semiconductor saturable absorber mirror (SESAM)^44^ with ≈1 ps recovery time. The combs are emitted from the laser cavity via an output coupler and subsequently coupled to individual single-mode fibers for beam delivery to the transceiver unit with about 70% efficiency. The performance characteristics are summarized in Table 1. The optical bandwidth after the fiber is broadened compared to the oscillator output due to self-phase modulation in the fiber.

**Table 1:** Dual-Comb Laser Parameters^a^

	unit	comb 1	comb 2
center wavelength	nm	1056	1055
*P*_average_ (oscillator)	mW	108	100
*P*_average_ (fiber)	mW	75	70
bandwidth (oscillator)	nm	14	10
bandwidth (fiber)	nm	22	17
*f*_rep_ (nominal)	GHz	1.041	
Δ*f*_rep_ (tuning range)	kHz	0–200	

a The average power *P*_average_ is specified for both the direct oscillator output and after the beam delivery fibers. Bandwidth refers to the full width at half maximum.

Intensity noise of the pump laser leads to timing jitter on the two combs. This issue was examined in detail for similar lasers before.^38,42^ In ref (38), correlated timing jitter fluctuations were obtained by splitting a single pump diode into two equal parts to pump each comb. Here, we adopt a different approach by using two pump diodes combined through a 50:50 fiber splitter so that each combs’ pump contains around half of the power from each diode. This results in an increased pump power while maintaining correlated pump power fluctuations, hence reducing the pump-power-induced effects on the timing jitter of Δ*f*_rep_.

The two low-noise combs emitted by the laser are coupled into FC/APC single-mode fibers inside the prototype housing to deliver the light to the transceiver unit, which will be discussed next.

### Dual-Comb Transceiver Unit

Our previous dual-comb ranging setup used a fiber-based transceiver unit,^34^ which enabled alignment-free operation while measuring the target distance with both combs simultaneously acting as signal comb and each other’s local oscillator, thereby enabling Vernier-based nonambiguity range unwrapping.^27^ An FC/PC output fiber was used to generate a reference reflection from its end face. However, we found that the fiber-based setup exhibited multiple etalon-like effects, leading to ghost pulses that can reduce the measurement precision when they are close to the target reflection. These effects arose from polarization projections due to imperfections in the fiber components and from a “cavity” formed between the output fiber tip and the target. Additionally, the reference reflection also led to a so-called dead zone^45^ when the target is an integer number of cavity lengths away from the fiber tip, as this causes the target and reference reflections to arrive at the same time and therefore interfere with each other. Such ghost-pulses and dead zones can limit the applicability of a ranging system for continuous measurements.

**Figure 2:**
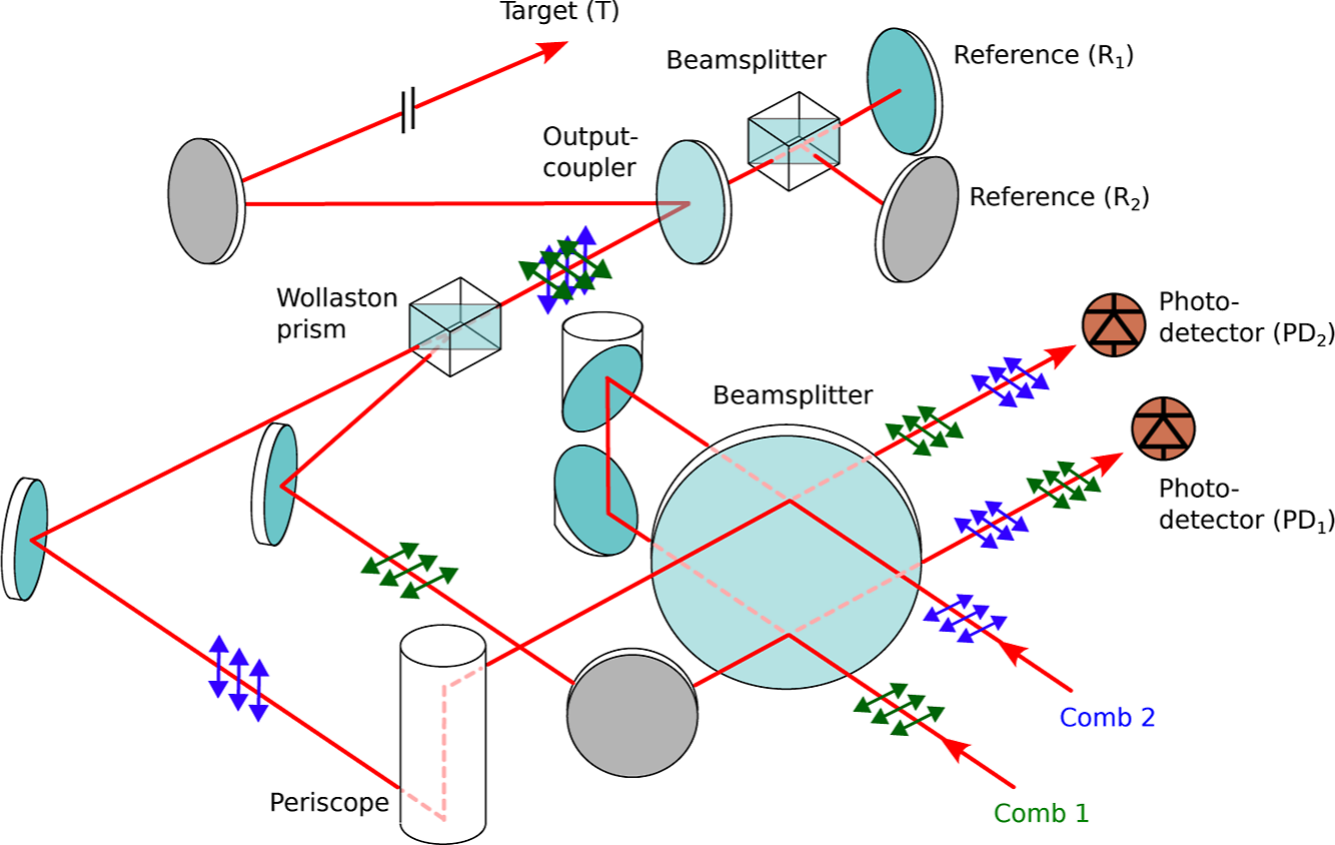
Schematic of the dual-comb transceiver unit with the polarization of comb 1 (comb 2) indicated in green (blue). The input polarization was adjusted with fiber polarization controllers on the delivery fiber from the laser source to the transceiver unit.

To address these issues, we developed a new setup that offers dead-zone-free measurements at any distance. Figure 2 shows a schematic of this new transceiver unit. Similar to the implementation in ref (34), it also allows measuring the target distance simultaneously with the roles of the combs interchanged. In particular, we combine the orthogonally polarized combs with a Wollaston prism and send them together through a polarization-maintaining (PM) single-mode fiber for mode-matching so that they are colinear along the measurement path. This ensures that both combs travel the same distance along the propagation path between the subsequent reference and target. Since the PM fiber is placed before this propagation path, the different group velocities along its two polarization axes do not affect the measured distance. On PD_*i*_ (*i* = 1, 2), we can then record the distance probed with comb *i*, while comb *j* (*j* = 2, 1) serves as local oscillator to map the optical signal pulses reflected by both references and target into the electronic time domain. This downconversion allows for easy detection of these signals with a photodiode (DET08C/M, Thorlabs) and subsequent electronic amplification (ENA220-T, RF-Bay), while maintaining the high measurement precision inherent to the optical time domain. We use the two measurements to deduce the number  of nonambiguity ranges *R*_A,i_ to the target, thereby extending the effective nonambiguity range to several kilometers using the Vernier effect. Furthermore, since these measurements are recorded simultaneously, we can perform this computation for each Δ*f*_rep_ period of the data, which allows tracking moving targets at long distances.^34^

**Figure 3:**
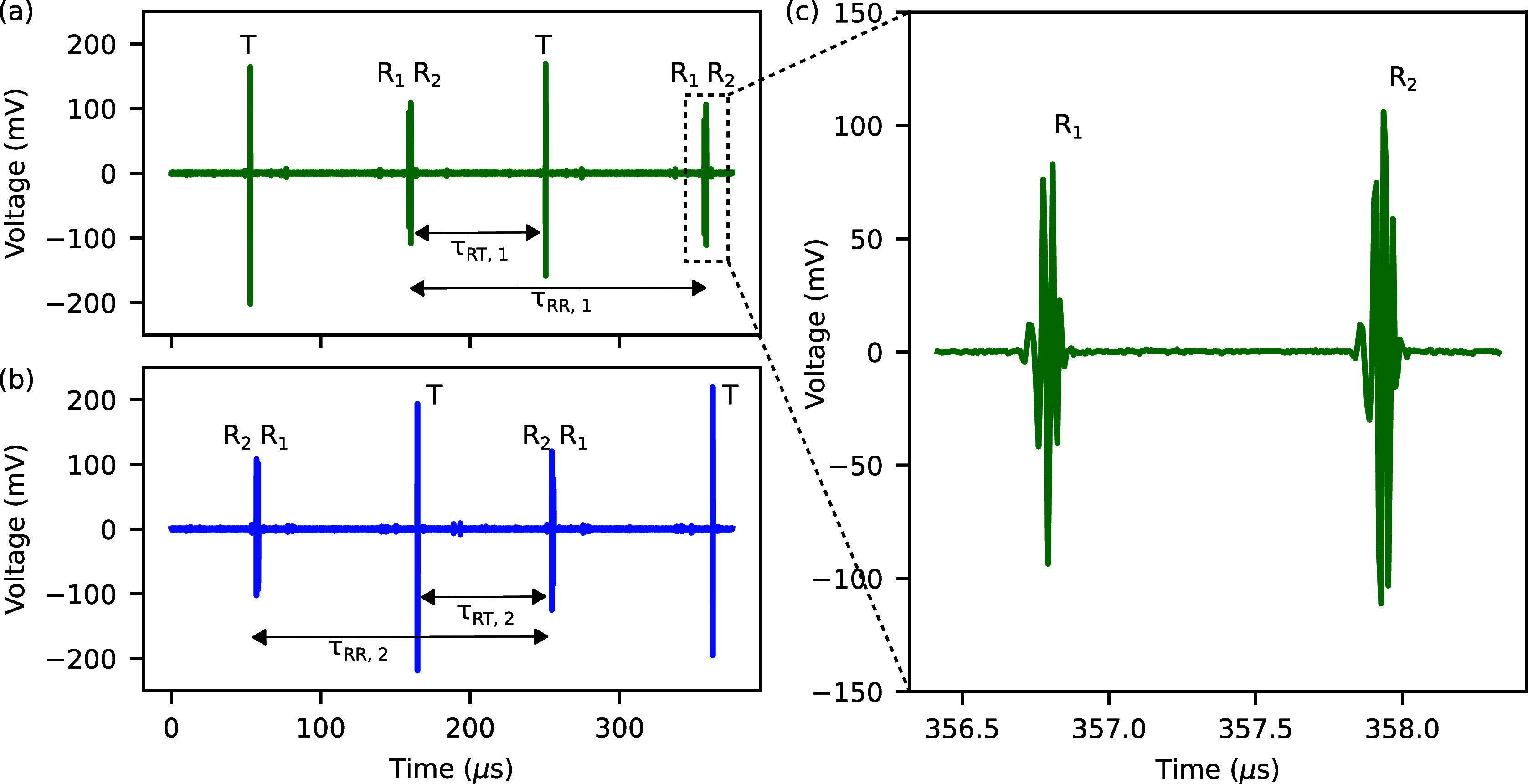
(a) Interferogram trace which shows the reference reflections (*R*_1_ and *R*_2_) and target reflection (*T*). The delay τ_RT,1_ encodes the separation between reference and target, while the delay τ_RR,1_ = 1/Δ*f*_rep_ = 198 μs indicates the measurement update rate. (b) Interferogram signal recorded on the second photodetector with the roles of signal beam and local oscillator interchanged. This simultaneously recorded second channel allows to extract the number of nonambiguity ranges on each measurement period. (c) Zoom on the two reference IGMs which allow for dead-zone-free measurements.

For certain targets, the signal pulses can be partially depolarized. This leads to a reduced IGM strength but does not distort the signal with spurious peaks.^34^ Furthermore, the transceiver unit mainly relies on free-space components to suppress unwanted reflections or polarization projections that lead to etalon-like features in the IGMs. Although such reflections are still present in our measurement, they are close to the noise floor as visible in Figure 3a,b, which shows typical interferogram traces. The IGM signals correspond to the light reflected by the target (*T*) and two references (*R*_1_ and *R*_2_). The reference reflections are generated outside the target path using an output coupler (Figure 2), as this prevents the formation of a cavity between the reference and target planes. The delay τ_RR,*i*_ = 1/Δ*f*_rep_ between consecutive pairs of signals from the same reference (i.e., *R*_1_ to *R*_1_ and *R*_2_ to *R*_2_) provides the repetition rate difference and the delay τ_RT,*i*_ between the target and reference reflections encodes the measurement distance. Thus, the absolute distance based on the time-of-flight information is
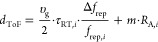
3

To avoid dead zones from the overlap of reference and target interferograms, the transceiver unit creates two reference reflections with a small offset relative to each other, as visible in Figure 3c. This ensures that there is always at least one reference not overlapping with the target IGM. In case the target IGM would overlap with one of the reference IGMs, i.e., if we are within the dead zone, the second (undistorted) reference can still be used to (i) get an accurate estimate of the reference position; (ii) subtract the distorted reference IGM to clean the target IGM and thereby enable dead-zone-free measurements as discussed in detail in the next section; and (iii) infer and correct for the laser timing fluctuations by tracking Δ*f*_rep_ via τ_RR,*i*_. To also account for fluctuations in *f*_rep_, e.g., a drift over time, we record the down-mixed product between the pulse repetition rates and a 1 GHz output of a signal generator, alongside the interferometric dual-comb signals, on our data acquisition card (M4i.4451-x8, Spectrum Instrumentation). The clocks of the data acquisition card and the signal generator are both synchronized to the same 10 MHz Rubidium atomic clock (FS725, Stanford Research Systems) with an accuracy of <4 × 10^–9^ and a short-term stability of <2 × 10^–12^ (100 s). This enables tracking of the absolute value of *f*_rep_, which helps to ensure long-term measurement stability.

### Data Analysis

Real-time phase- and timing correction of the dual-comb interferograms is a critical aspect for obtaining large data sets, long integration times, or deploying such systems to real-world measurements. Two of the most popular strategies are solutions based on field-programmable gate arrays (FPGAs)^46^ and GPUs.^35,36^ FPGAs are advantageous in terms of low power consumption and low latency, but GPUs are more user-friendly, as they can be programmed with standard programming interfaces such as CUDA (as opposed to the hardware description tools necessary for FPGA development). There are several data acquisition cards with established interfaces to the GPU, making this a generally accessible approach. Here we use a GPU approach based on the M4i.4451-x8 digitizer card (Spectrum Instrumentation), operating at a sampling rate of 125 MS/s. Our approach is conceptually similar to those discussed elsewhere but adapted to the particular data processing steps involved with the new transceiver unit.

**Figure 4:**
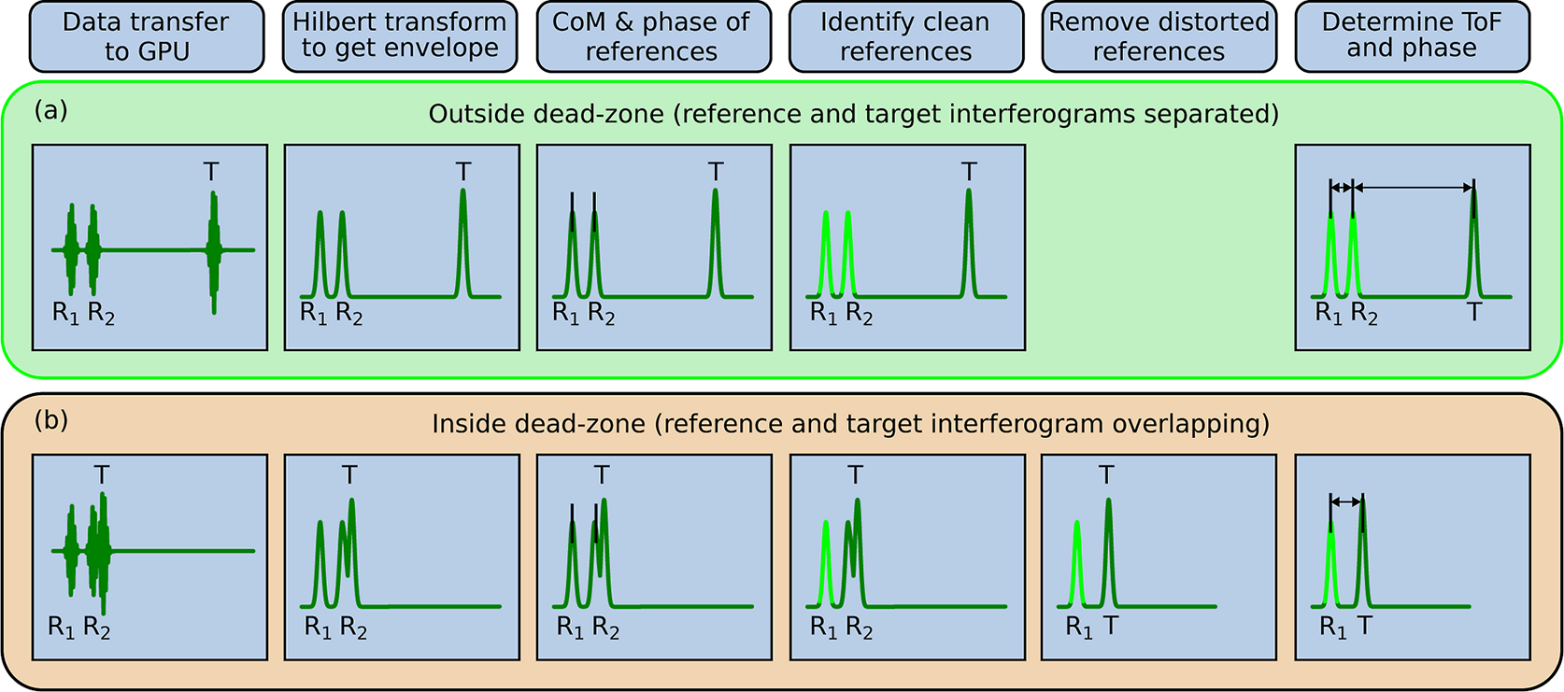
Relevant processing steps of the GPU-accelerated algorithm for the time-of-flight and phase-based distance measurement (a) outside the dead zone and (b) within the dead zone. The clean references, i.e., those not overlapping with the target IGM, are indicated in light green. *T*: target interferogram, *R*_*i*_: reference interferograms, CoM: center-of-mass.

An overview of the data processing steps performed by the real-time GPU algorithm is shown in Figure 4. We distinguish between the cases outside the dead zone, i.e., when the reference and target IGMs do not overlap (Figure 4a), and within the dead zone, i.e., when the target IGM is distorted (Figure 4b). In both cases, the data are first transferred to the GPU, followed by a frequency-shift of the dual-comb signal toward DC and a Hilbert transform to extract the envelope. We then compute (i) the phase delay between the two references *R*_1_ and *R*_2_, (ii) the phase delay to the corresponding references in the previous period, and (iii) the center-of-mass of the references using a second-order moment integral where we additionally filter out signals below a certain threshold to be less susceptible to ghost pulses.

To allow for a comparison between references, we then use this information to compute the difference between the reference IGMs by subtracting one interferogram from the other, while accounting for the measured difference in their amplitude and phase. By comparing the two references *R*_1_ and *R*_2_ with each other and to the corresponding reference in the previous period, we can determine if one of them overlaps with the target and also identify which reference overlaps if there is one. The references not overlapping with the target IGM are marked as clean references.

**Figure 5:**
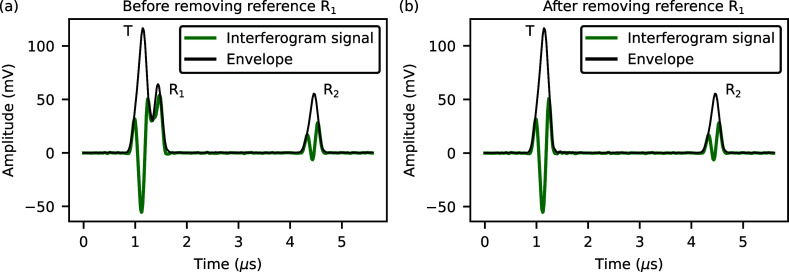
(a) Example trace showing the situation of overlapping target and reference IGMs (here *R*_1_ is overlapping with *T*), which was extracted during the processing of the actual measurement data. (b) By subtracting reference *R*_2_ from *R*_1_, while accounting for the measured difference in their position, amplitude, and phase, we suppress *R*_1_ and thereby clean the target IGM to avoid systematic errors in the center-of-mass estimation caused by their overlap.

The center-of-mass and phase-delay computed for the clean references allows us to update the measurement parameters such as, e.g., Δ*f*_rep_, and clean the target IGM in case it overlaps with a reference by subtracting the clean reference from the distorted reference as illustrated in Figure 5. For this subtraction procedure, we account for the measured difference in the position, amplitude, and phase between the references (based on the most recent measurement where both references were clean). Finally, we obtain clean reference and target IGMs from which we can calculate the time-of-flight and phase delay required for extracting the distance information. To make the Vernier-based nonambiguity range extension more robust against small fluctuations in the measured ToF-distance, we calculate the number *m* of nonambiguity ranges with an exponential moving average and restrict changes to ±1 from one period to the next.

It is important that the algorithm achieves a sufficiently high throughput: if it is too low, then Δ*f*_rep_ must be reduced accordingly to allow for real-time processing, and eventually, this precludes fully coherent measurements from a free-running dual-comb laser. In our case, the full algorithm (including both ToF and phase extraction) could be implemented at up to around 7.1 kHz. A limiting factor of the processing time is the Hilbert transform, which requires the computation of two FFTs. In our case, this step alone can take up to ≈100 μs on our GPU (RTX A5000, NVIDIA) when working with the single-precision floating point format (FP32). On another PC equipped with an Intel Core i7–9700K CPU and 32 GB RAM, the Hilbert transform takes a longer processing time of around 1 ms, which shows the advantages of GPU-based calculations. To further keep the processing time of each period consistent and as short as possible, the algorithm is designed to only use element-wise and reduction operations such as summations or maximum finding, rather than relying on iterative processes such as curve fitting.

Because the new laser employs a robust prototype cavity construction (Figure 1), it is compatible with coherent averaging down to Δ*f*_rep_ below 1 kHz. This enables fully coherent data processing with the GPU-accelerated algorithm. To operate between these limitations imposed by coherent averaging capability and GPU-processing speed and to also balance update rate with the effective number of detected signal photons per period *N*_photon_ ∝ 1/Δ*f*_rep_ which is beneficial for the measurement precision (see eq 1), we chose a repetition rate difference of Δ*f*_rep_ = 5.06 kHz. These considerations also highlight that, when targeting high update rate measurements with free-running dual combs, it is important to consider both the constraints imposed by the laser and those related to data processing. Note that to avoid spectral aliasing, there are additional constraints on the position of the IGMs in the RF domain and thus the update rate. As a result, not all Δ*f*_rep_ settings are actually viable in the case that Δ*f*_CEO_ is not controllable.

For the data analysis presented in the following section, we recorded several long time traces of ≈500 s and used them as an input for our real-time processing code. This allowed us to refine and optimize our algorithm “offline” using one of the traces before ultimately applying the algorithm to process another trace to obtain the data shown in this paper. Here we confirmed real-time processing capability by ensuring a processing rate faster than Δ*f*_rep_: the processing of each period takes ≈140 μs corresponding to the aforementioned maximum update rate of 7.1 kHz.

## Results

### Measurement Bench

**Figure 6:**
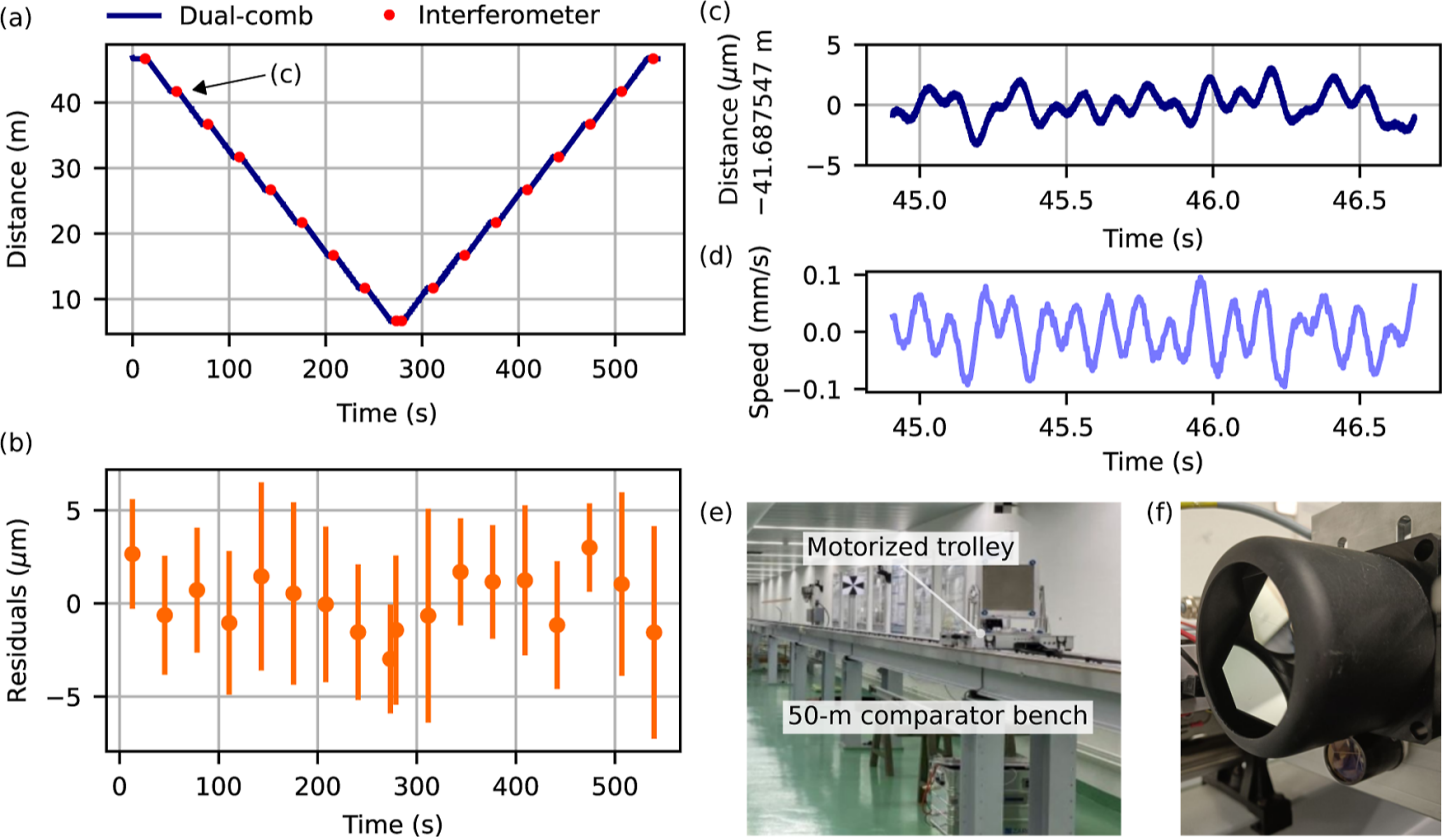
(a) Measurements obtained with the dual-comb ranging setup (using the time-of-flight information), compared with reference measurements from a Doppler interferometer. (b) Residuals of the dual-comb measurement with respect to the interferometric measurement: mean (dot) and maximum variation (error-bars) represent the slow oscillations across the static regions. (c) Measured distance during the static region at around 45 s, which exhibits slow oscillations and (d) corresponding velocity. (e) Image of the 50 m comparator bench used for the long-distance measurements and (f) the two retroreflectors used for the dual-comb ranging (top) and reference interferometer (bottom).

We conducted our experiments in controlled laboratory conditions on a 50 m long linear comparator bench (see Figure 6e). The comparator bench is equipped with a computer-controlled motorized trolley that could move along its entire length. The bench further includes a Doppler interferometer based on a helium–neon laser (HP5519A, Keysight) to take calibrated snapshots of the distance. Our dual-comb apparatus was positioned next to the bench, and we directed the combs emitted by the transceiver unit to an optical breadboard mounted on the bench to launch them toward the target. On the breadboard, the beams are first expanded and then collimated with a pair of lenses forming a Keplerian telescope to prevent significant beam divergence along the measurement path. This telescope is also used to collect back-reflected light. The combs were aligned parallel to the reference interferometer beam along the measurement path. The target is a retroreflector mounted on the movable trolley. There is also a second retroreflector (mounted below the target retroreflector) for the reference measurement with the interferometer (see Figure 6f).

Since there is a nonzero baseline between these retroreflectors (≈5.6 cm), small tilts of the trolley caused, e.g., by bending of the rails introduces an offset between the distance measured with the two systems. This offset is known as Abbé-error. To estimate this offset, we measured the inclination of the trolley with an inclination sensor (065–040TYPE3–10, Zerotronic) as it moved along the comparator bench. Together with the baseline between the retroreflectors, we then approximate the Abbé-error and correct it in the reference measurement.

Our objectives for the measurement campaign included demonstrating real-time measurements, maintaining absolute calibration over long distances (here demonstrated over a 40 m range), conducting continuous measurements for several minutes, and enhancing the precision by exploiting the IGM phase information for interferometric measurements. To show these capabilities, the trolley was initially positioned at around 50 m and was automatically moved closer in 5 m steps to around 10 m, remaining stationary at each intermediate position for 5 s, as depicted in Figure 6a. At each step, we recorded a reference measurement of the distance with a Doppler interferometer. Similarly, the trolley was then moved again to around 50 m in 5 m increments to evaluate any systematic deviations relative to the reference measurements and to assess the temporal stability of the experimental setup. The software controlling the stop-and-go movement of the trolley outputs a single interferometer distance reading after the trolley stops at each location, with time stamps accurate to within a few 0.1 s. We therefore only used the interferometer readings to check for systematic offsets in the absolute distance measurement at the stationary trolley positions. The reference measurement accounts for the phase refractive index in air at approximately 633 nm (helium–neon laser). We also scale the ToF-based dual-comb ranging measurement with the inverse of the group refractive index *n*_g_ at λ_c_ calculated from the corrected Ciddor’s equation provided in ref (47) using the meteorological parameters continuously monitored in the lab.

We launch around 1 mW of optical power per comb toward the retroreflector target. The distance to the target affects the coupling efficiency of the back-reflected light into the PM single-mode fiber and thus the power on the photodetectors. Typically, it is tens of μW per comb.

### Accuracy

For each stationary trolley position, we compare the mean of the ToF-based dual-comb ranging measurements during this static region to the reference measurement obtained from a Doppler interferometer. Since the Doppler interferometer measures the distance relative to the initial trolley position, these reference measurements were aligned to the absolute dual-comb ranging measurements by adding a global distance offset. From Figure 6b we find that the residuals between the dual-comb ranging and shifted Doppler interferometer measurements are below 3 μm across the 40 m range. The error bars in Figure 6b represent the variation of our dual-comb ToF-based distance measurements across the different static windows at each position. They are dominated by a slow oscillation caused by external instabilities resulting in real or apparent distance changes with amplitudes on the order of a few μm and a dominant frequency of around 10 Hz. This is illustrated in Figure 6c for the measurement at the static position, where the trolley arrives after around 45 s. Figure 6d shows the corresponding velocity determined from the ToF data after application of a low-pass filter to extract the slow oscillation.

Without, e.g., tracking the velocity, the nonambiguity range extension is only possible up to a delay of Δτ = 0.5Δ*f*_rep_/*f*_rep_^2^ from one period to the next. In our case, this corresponds to a maximum target speed of roughly 1.77 mm/s. As apparent from Figure 6d, it would thus not be necessary to track the target speed within the static regions. However, to also enable accurate measurements when the trolley is moving between the static regions, our system accounts for the target’s speed by interpolating the distance measurements to a common reference time between the channels.^34^ When tracking and correcting for velocity, a more general limit exists also with respect to acceleration.

### Precision

Next, we consider the precision of the ToF-based distance measurement. Since the available reference measurements undersample the motion of the target, we need an alternative means to infer the precision of the dual-comb ranging measurement. For that purpose, we consider short time windows of 5 periods (=5/Δ*f*_rep_ ≈ 1 ms), compute a linear fit through the measurements within each time window, and calculate the standard deviation of the resulting residuals compared to the fit to quantify the precision. To further account for the dependence of precision on the relative delay of reference and target interferograms,^48^ these time windows are from measurements where the target is moving between two static regions.

**Figure 7:**
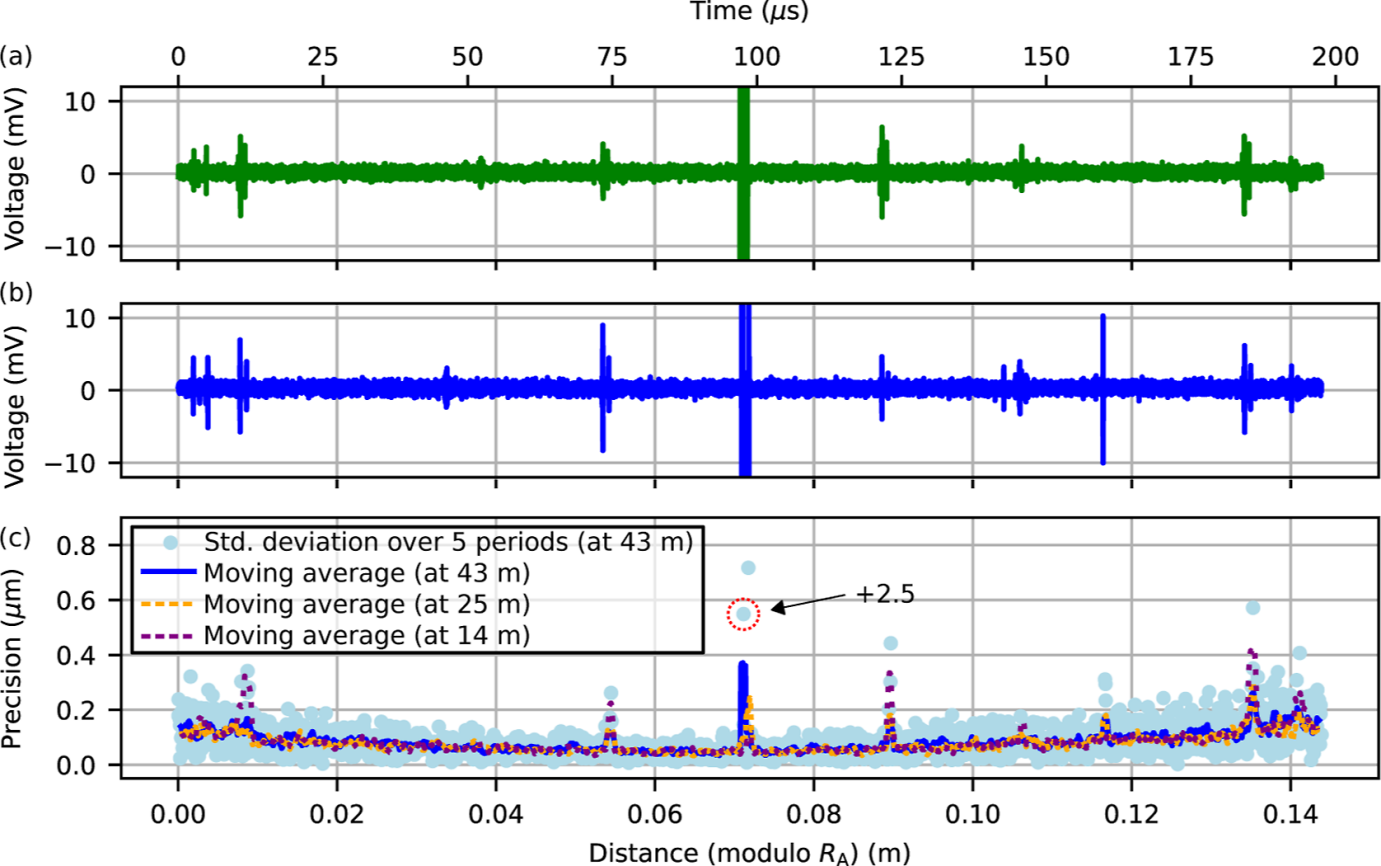
Vertical zoom on one period of the IGM trace recorded with (a) the first photodetector (PD_1_) and (b) the second photodetector (PD_2_) with inverted time-axis. These example traces were selected and aligned such that the target (*T*) and reference (*R*) IGMs are both located at the center of the window. (c) Ranging precision for different offsets between *T* and *R*, calculated from the standard deviation of 5 distance measurements w.r.t. their linear fit for measurements at around 43 m where the target is moving so that the delay between *T* and *R* changes. This delay τ_RT_ is encoded in the distance υ_g_/2·τ_RT_ (modulo the nonambiguity range, *R*_A_). The highlighted point has been shifted down by 2.5 μm to keep the rest of the features visible. We also compute a moving average (window size of 10 points) over the measurements around 43 m (blue), 25 m (orange), and 14 m (purple).

More specifically, since the target is moving, the precision measured for the individual time windows corresponds to different absolute distances and thus different delays between target and reference IGMs. Generally, shorter delays between reference and target lead to reduced relative timing jitter and hence improved precision.^48^ To account for this, we sort the measurements according to the absolute distance modulo the nonambiguity range *R*_A,*i*_. This analysis routine was performed for regions around 43, 25, and 14 m, which all indicate similar precision as shown in Figure 7c. The center of the plot corresponds to the measurements when the target IGM overlaps with the reference IGMs, and the edges of Figure 7c correspond to the situation where the target IGM is furthest away from the reference IGMs on the acquired electronic signal. For the latter case, the relative timing jitter between reference and target is highest,^48^ which explains the degradation of precision toward the edges in Figure 7c. For most of the measurements, the point-to-point fluctuations have a standard deviation of <0.1 μm. At a few specific positions, the precision degrades from ≪1 to ≈1 μm.

In Figure 7c, the degradation in the center originates from the reference IGMs. Using a pair of reference reflections allows the subtraction of the reference part of the waveform when it overlaps with the target reflection (see Figure 5). However, this subtraction is not always perfect and introduces noise of the subtracted signal into the resulting signal, potentially reducing the precision at those positions. Alternative approaches to circumvent this issue include separating the reference and target pulses based on their polarization^45,49^ or having the reference pulses on a separate optical path, i.e., preventing them from propagating back along with the target reflection. While these approaches avoid the issue of overlapping IGMs, they require additional detectors and data channels, increasing the complexity of the required electronics. Moreover, using a separate path for the reference pulses may reduce the measurement accuracy due to systematic offsets between the reference and signal paths (as this configuration lacks the near-common-path architecture).

The remaining positions for which the precision degrades in Figure 7c originate from ghost pulses, which are revealed when zooming in on the vertical axis of the dual-comb signals recorded with the two photodetectors. To visualize this, we selected and aligned example traces recorded with the first photodetector (Figure 7a) and second photodetector (Figure 7b, with an inverted time-axis) to the measurements in Figure 7c such that the reference and target IGMs are in the center. This illustrates that while the free-space setup can significantly reduce ghost-pulses compared to the fiber-based implementation of the transceiver unit,^34^ such ghost pulses still remain a limiting factor that causes degradation of the precision at certain measurement distances.

### Interferometric Hand-Over

Next, we investigate the phase information in the IGMs, which has the potential to significantly improve the measurement precision.^27^ Since our dual-comb IGMs are sufficiently stable for coherent averaging in free-running operation at the selected update rate, phase information can be tracked unambiguously as long as (i) the target does not move faster than λ_c_/4·Δ*f*_rep_ to prevent ambiguity in unwrapping the phase between subsequent IGMs and (ii) the Doppler-induced phase change between successive target IGMs due to the target’s acceleration is smaller than π. More specifically, the dual-comb IGMs encode the instantaneous optical transfer function (OTF) associated with the measurement setup, i.e., the spectral phase associated with free space propagation to the target and back. However, since we are using free-running dual combs with unknown carrier-envelope offset frequency *f*_CEO_ and unknown comb line indices, there is an unknown offset in mapping the measured RF comb line frequencies to the corresponding optical comb line frequencies. While this precludes recovery of the true OTF, the optical frequencies are still known reasonably well due to the known center wavelength measured with an optical spectrum analyzer. Therefore, if we utilize the ToF data to calibrate the absolute optical delay associated with a particular target position, small distance changes around that position can be inferred by the phase instead, thereby improving the precision.

To achieve this, we use our GPU-accelerated algorithm to compute and unwrap the phase delay from the reference to the target IGM alongside the ToF information. Since the resulting phase ϕ(*t*) is proportional to the center wavelength (up to some offset influenced by *f*_CEO_), we can employ these phase changes to track relative distance changes with interferometric precision. To get absolute distance measurements with interferometric precision, we can use the ToF information to shift the interferometric distance measurement once by *d*_ToF_ and then continue tracking the distance based on the interferometric phase information, resulting in
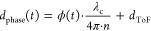
4where *n* is the phase refractive index at λ_c_, which can be estimated from empirical equations, e.g., Ciddor’s equation.^50^ Alternative schemes for the interferometric hand-over rely on counting the number of optical wavelengths based on the ToF measurement to achieve interferometric precision combined with absolute distance measurement. While our ToF precision would be sufficient for this approach (ToF precision below λ_c_/4), we lack information about the absolute phase offset due to not being tracked and free-running *f*_CEO_.

Furthermore, there can be a small error in the determination of ϕ(*t*) if the carrier frequency of the interferograms is not shifted exactly to DC. This error is on the order of <10 mrad and therefore does not limit the use of the interferometric phase to track local changes in the target position. When interferometrically tracking the target across larger distances, it would be necessary either to determine the carrier frequency by calibration or to reset the distance measurement using the ToF measurements. Similarly, the ToF information could also be used to relax the limits on the target’s speed and acceleration for interferometric ranging: using the absolute distance information from the ToF-based measurement, it is possible to account both for the Doppler shift and for phase changes larger than ± π between subsequent IGMs due to the target’s motion.

**Figure 8:**
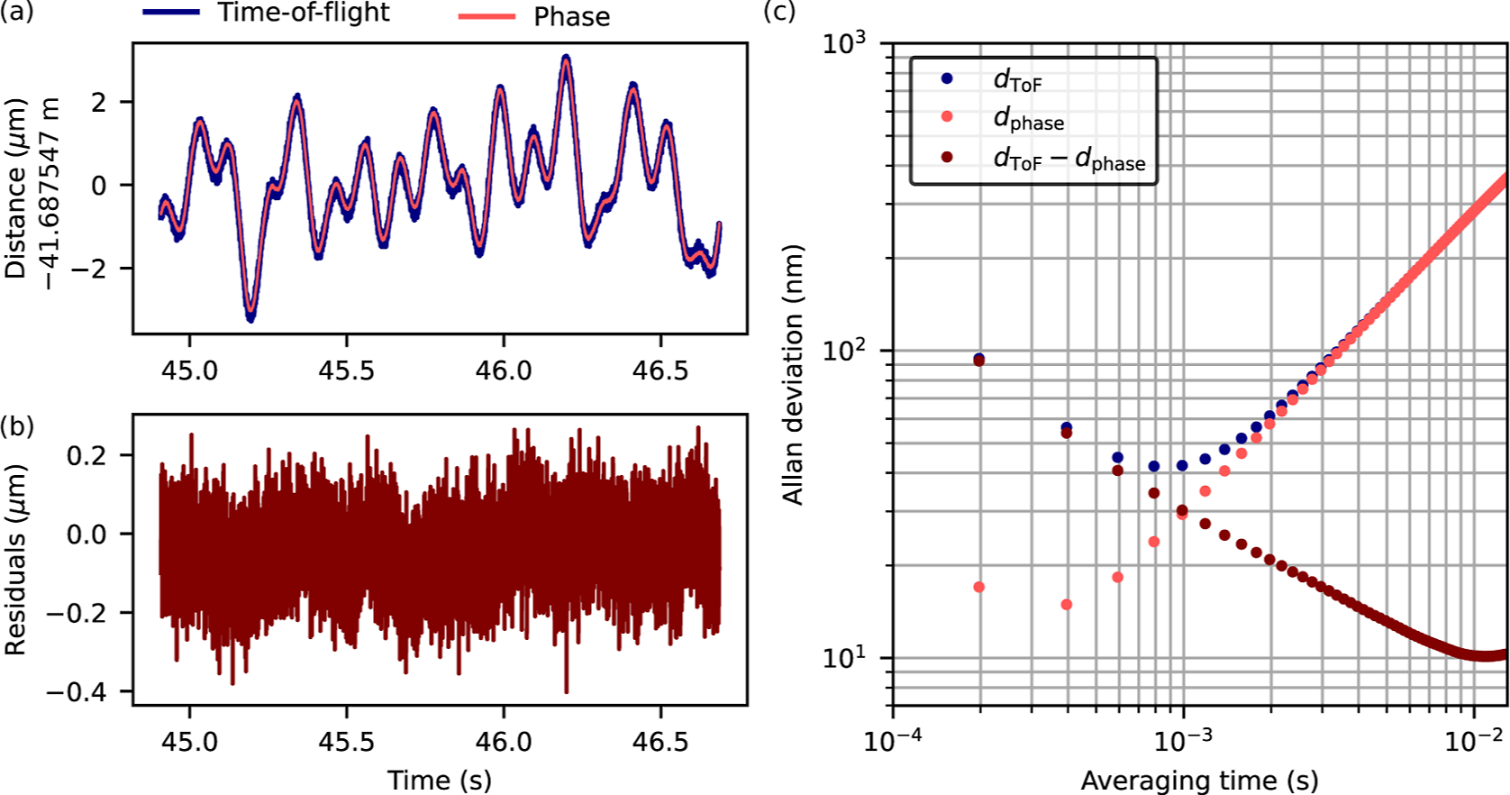
(a) Time-of-flight (blue) and phase-based (pink) distance measurement during the static region at around 45 s. (b) Residuals between the time-of-flight (ToF) and phase-based distance measurements. (c) Allan deviation for the distance measurement based on ToF (*d*_ToF_, blue) and phase (*d*_phase_, pink) information. It also shows the Allan deviation for the residuals in (b), i.e., for *d*_ToF_ – *d*_phase_, which indicates the ToF-precision without the influence of the slow oscillation on a 100 ms time scale.

In Figure 8a, we examine the phase-based interferometric measurements overlaid with the ToF measurement for the static-target segment at around 45 s, as shown previously in Figure 6c. The phase-based distance measurements (in pink) are in close agreement with the ToF-based measurements (in blue). The residuals between the two measurements are shown in Figure 8b.

As apparent from the Allan deviation in Figure 8c, the interferometric hand-over significantly improves the single-shot precision from around 0.1 μm for the ToF-based measurement (blue) down to less than 20 nm for the interferometric measurement (pink). Averaging improves the measurement precision at first, but after an averaging time of around 1 ms, the precision starts degrading due to the slow oscillation. To compensate for the precision degradation due to this slow oscillation, we can also consider the Allan deviation for the residuals in Figure 8b. Due to the high precision of the interferometric measurement, this can serve as an indicator for the true precision achievable with the ToF-based measurement under suitable conditions (vacuum, stable setup), which reaches around 10 nm after averaging for 10 ms.

## Conclusions

We demonstrate a ranging system based on a free-running single-cavity Yb:CaF_2_ 1-GHz dual-comb laser combined with a free-space transceiver unit optimized to suppress problematic spurious ghost pulses. The system allows for simultaneous distance measurements with the role of the two combs interchanged to track moving targets even at a long-range.^34^ By generating two reference reflections with a short delay relative to each other, the setup can further circumvent dead zones caused by regions where the target and reference IGMs overlap. To additionally exploit the fast measurement update rate of 5.06 kHz, we developed a GPU-accelerated algorithm that enables real-time data processing.

The performance of the dual-comb ranging instrument is tested on a 50 m long comparator bench equipped with a cooperative target placed on a movable trolley. Comparison to a reference interferometer suggests an accuracy of around ±3 μm for repeated measurements every 5 m over 40 m. We achieve a single-shot time-of-flight precision of around 0.1 μm, i.e., below λ_c_/4. Due to the laser’s low-noise properties (inherent to the prototype housing and low-pass filtering of pump noise), we can track the phase delay between reference and target IGMs even in free-running operation. This allows for interferometric distance tracking, which yields a single-shot precision below 20 nm.

The reported accuracy and precision indicate the achievable performance in a controlled laboratory environment, where the meteorological parameters were continuously monitored to calculate the refractive index of air. In practical outdoor conditions, the achievable measurement accuracy is affected by spatial and temporal variations in the refractive index of air along the measurement path, resulting in apparent distance errors. Compensating for these errors requires monitoring meteorological parameters at multiple locations along the measurement path^51^ or using simultaneous distance measurements at two or more wavelengths for dispersion-based refractivity correction.^52^

Our results showcase the potential of the proposed dead-zone-free dual-comb ranging approach for applications requiring real-time and accurate long-distance measurements in controlled environments such as industrial process monitoring as well as in space applications, including satellite positioning and formation flying.

## Supporting Information Available

Derivation of the CRLB for time-of-flight measurements, which was used in eq 1, and simulation of the intracavity beam evolution (PDF)
