# Supplementary material for: Long-Range and Dead-Zone-Free Dual-Comb Ranging for the Interferometric Tracking of Moving Targets
Source: ACS Photonics. 2025 Mar 21;12(4):1829–39. doi: 10.1021/acsphotonics.4c02199 (PMC12007099; doi:10.1021/acsphotonics.4c02199)
Supplement: Supplementary file 1 — ph4c02199_si_001.pdf [file ph4c02199_si_001.pdf]

Supporting Information: Long-range and dead-zone free dual-comb ranging for the interferometric tracking of moving targets

Sandro L. Camenzind,[*,†,¶] Lukas Lang,[†,¶] Benjamin Willenberg,[†] Justinas Pupeikis,[†] Hayk Soghomonyan,[†] Robert Presl,[‡] Pabitro Ray,[‡] Andreas Wieser,[‡] Ursula Keller,[†] and Christopher R. Phillips[†]

†*Department of Physics, Institute for Quantum Electronics, ETH Zurich, 8093 Zurich, Switzerland*

‡*Department of Civil, Environmental and Geomatic Engineering, Institute of Geodesy and Photogrammetry, ETH Zurich, 8093 Zurich, Switzerland*

¶*These authors contributed equally to this work*

E-mail: casandro@phys.ethz.ch

Cramer-Rao lower bound for time-of-flight measurements

The Cramer-Rao lower bound (CRLB) for a ranging measurement subject to white Gaussian noise is discussed in Ref. S1. The lower bound on the variance on the inferred timing is given by

$$\sigma^2_{\mathrm{ToF}}(T_0) = \frac{\sigma^2_{\mathrm{WN}}}{2B\int \left(\frac{ds(t)}{dt}\right)^2 dt} \tag{1}$$

for additive white noise with variance σ^2_{WN}, and a signal $s(t)$ with a full bandwidth $2B$ around which the waveform is filtered to avoid unnecessary noise contributions from other frequencies.

We can adapt this formula to the case of the positive Fourier frequency branch of a dual-comb interferogram with shot noise limited measurement noise. For simplicity we make the assumption that the local oscillator current i_{LO} dominates the signal current i_{signal}, so that the one-sided shot noise current power spectral density (PSD) is $2qi_{\mathrm{LO}}$ with the elementary charge q. Furthermore, we assume that the IGM signal is given by the following expression:

$$\begin{aligned}\bar{s}(t) =& 2\sqrt{i_{\mathrm{LO}} i_{\mathrm{signal}}} \cdot \mathrm{sinc}\left(\frac{t}{T_{\mathrm{signal}}}\right)\cos(2\pi i\nu t)\\ =& s(t)\left(e^{2\pi i\nu t} + e^{-2\pi i\nu t}\right),\end{aligned} \tag{2}$$

where ν is the optical frequency, T_{signal} determines the IGM width, and the second expression introduces the signal $s(t) = \sqrt{i_{\mathrm{LO}} i_{\mathrm{signal}}} \cdot \mathrm{sinc}(t/T_{\mathrm{signal}})$. Since $s(t)$ corresponds to the positive Fourier branch, it has to be compared to the two-sided shot noise PSD qi_{LO}. We therefore substitute $\sigma^2_{\mathrm{WN}} = qi_{\mathrm{LO}} \cdot 2B$, and $s(t)$ as defined above into Eq. 1. This yields

$$\sigma^2_{\mathrm{ToF}}(T_0) = \frac{3}{\pi^2}\frac{qT_{\mathrm{signal}}}{i_{\mathrm{signal}}}. \tag{3}$$

This value corresponds to "oscilloscope time". To get the corresponding value for optical delay, the variance has to be multiplied by the scale factor $(\Delta f_{\mathrm{rep}}/f_{\mathrm{rep}})^2$. By additionally noting that the measurement time is $T_{\mathrm{meas}} = 1/\Delta f_{\mathrm{rep}}$ and that the full optical bandwidth is given by $\Delta\nu_{\mathrm{opt}} = 1/T_{\mathrm{signal}} \cdot f_{\mathrm{rep}}/\Delta f_{\mathrm{rep}}$, we find

$$\begin{aligned}\sigma^2_{\mathrm{ToF,opt}}(\tau_0) =& \frac{3}{\pi^2}\frac{1}{f_{\mathrm{rep}}\Delta\nu_{\mathrm{opt}}}\frac{q}{i_{\mathrm{signal}}T_{\mathrm{meas}}}\\ =& \frac{3}{\pi^2}\frac{1}{f_{\mathrm{rep}}\Delta\nu_{\mathrm{opt}}N_{\mathrm{photon}}},\end{aligned} \tag{4}$$

where an effective number of detected signal photons $N_{\mathrm{photon}} = i_{\mathrm{signal}} T_{\mathrm{meas}}/q$ has been introduced. The prefactor $3/\pi^2$ depends on the pulse shape, so for the inequality in Eq. 1 of the main text we have set it to $O(1)$ to make the result more general.

Simulation of the intra-cavity beam evolution

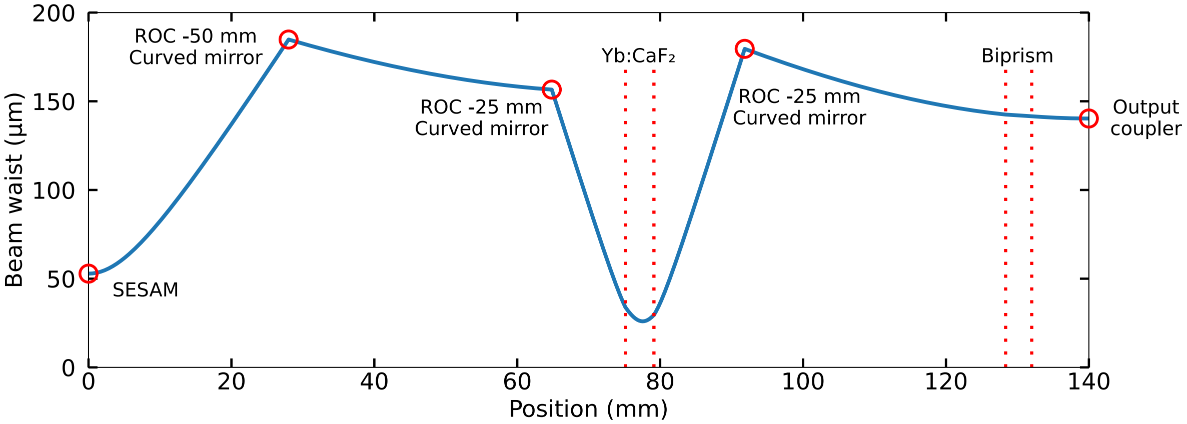

Figure S1: Simulation of the $1/\mathrm{e}^2$ intensity beam radius of the intra-cavity mode. The position of the relevant optical elements are marked with a red circle. The radius of curvature (ROC) of the curved mirrors is specified.

References

(S1) Kay, S. M. *Fundamentals of Statistical Signal Processing, Volume 1: Estimation Theory*; Pearson Education, 1993.
